# No association for Chinese HBV-related hepatocellular carcinoma susceptibility SNP in other East Asian populations

**DOI:** 10.1186/1471-2350-13-47

**Published:** 2012-06-19

**Authors:** Hiromi Sawai, Nao Nishida, Hamdi Mbarek, Koichi Matsuda, Yoriko Mawatari, Megumi Yamaoka, Shuhei Hige, Jong-Hon Kang, Koichi Abe, Satoshi Mochida, Masaaki Watanabe, Masayuki Kurosaki, Yasuhiro Asahina, Namiki Izumi, Masao Honda, Shuichi Kaneko, Eiji Tanaka, Kentaro Matsuura, Yoshito Itoh, Eiji Mita, Masaaki Korenaga, Keisuke Hino, Yoshikazu Murawaki, Yoichi Hiasa, Tatsuya Ide, Kiyoaki Ito, Masaya Sugiyama, Sang Hoon Ahn, Kwang-Hyub Han, Jun Yong Park, Man-Fung Yuen, Yusuke Nakamura, Yasuhito Tanaka, Masashi Mizokami, Katsushi Tokunaga

**Affiliations:** 1 Department of Human Genetics, Graduate School of Medicine, The University of Tokyo, 7-3-1 Hongo, Bunkyo-ku, Tokyo, 113-0033, Japan; 2 The Research Center for Hepatitis and Immunology, National Center for Global Health and Medicine, Ichikawa, Japan; 3 Laboratory of Molecular Medicine, Human Genome Center, Institute of Medical Science, The University of Tokyo, Tokyo, Japan; 4 Department of Internal Medicine, Hokkaido University Graduate School of Medicine, Sapporo, Japan; 5 Department of Internal Medicine, Teine Keijinkai Hospital, Sapporo, Japan; 6 First Department of Internal Medicine, Iwate Medical University, Iwate, Japan; 7 Division of Gastroenterology and Hepatology, Internal Medicine, Saitama Medical University, Saitama, Japan; 8 Department of Gastroenterology, Kitasato University School of Medicine, Sagamihara, Kanagawa, Japan; 9 Division of Gastroenterology and Hepatology, Musashino Red Cross Hospital, Tokyo, Japan; 10 Department of Gastroenterology, Kanazawa University Graduate School of Medicine, Kanazawa, Japan; 11 Department of Medicine, Shinshu University School of Medicine, Matsumoto, Japan; 12 Department of Clinical Molecular Informative Medicine, Nagoya City University Graduate School of Medical Sciences, Nagoya, Japan; 13 Molecular Gastroenterology and Hepatology, Kyoto Prefectural University of Medicine, Kyoto, Japan; 14 National Hospital Organization Osaka National Hospital, Osaka, Japan; 15 Division of Hepatology and Pancreatology, Kawasaki Medical College, Kurashiki, Japan; 16 Second department of Internal Medicine, Faculty of Medicine, Tottori University, Yonago, Japan; 17 Department of Gastroenterology and Metabology, Ehime University Graduate School of Medicine, Ehime, Japan; 18 Division of Gastroenterology, Department of Medicine, Kurume University School of Medicine, Fukuoka, Japan; 19 Department of International Medicine, Yonsei University College of Medicine, Seoul, Korea; 20 Department of Medicine, the University of Hong Kong, Queen Mary Hospital, Hong Kong, China

**Keywords:** Hepatitis B, hepatocellular carcinoma, candidate SNP, replication study, genome-wide association study

## Abstract

**Background:**

A recent genome-wide association study (GWAS) using chronic HBV (hepatitis B virus) carriers with and without hepatocellular carcinoma (HCC) in five independent Chinese populations found that one SNP (rs17401966) in *KIF1B* was associated with susceptibility to HCC. In the present study, a total of 580 HBV-derived HCC cases and 1351 individuals with chronic hepatitis B (CHB) or asymptomatic carrier (ASC) were used for replication studies in order to evaluate the reported association with HBV-derived HCC in other East Asian populations.

**Results:**

We did not detect any associations between rs17401966 and HCC in the Japanese cohorts (replication 1: OR = 1.09, 95 % CI = 0.82-1.43; replication 2: OR = 0.79, 95 % CI = 0.54-1.15), in the Korean cohort (replication 3: OR = 0.95, 95 % CI = 0.66-1.36), or in the Hong Kong Chinese cohort (replication 4: OR = 1.17, 95 % CI = 0.79-1.75). Meta-analysis using these cohorts also did not show any associations with P = 0.97.

**Conclusions:**

None of the replication cohorts showed associations between rs17401966 and HBV-derived HCC. This may be due to differences in the genetic diversity among the Japanese, Korean and Chinese populations. Other reasons could be the high complexity of multivariate interactions between the genomic information and the phenotype that is manifesting. A much wider range of investigations is needed in order to elucidate the differences in HCC susceptibility among these Asian populations.

## Background

Hepatitis B (HB) is a potentially life-threatening liver infection caused by the hepatitis B virus (HBV), and approximately 360 million people worldwide are thought to be chronically infected with HBV. The clinical course of HBV infection is variable, including acute self-limiting infection, fulminant hepatic failure, inactive carrier state and chronic hepatitis with progression to cirrhosis and hepatocellular carcinoma (HCC). Although some HBV carriers spontaneously eliminate the virus, 2-10 % of individuals with chronic HB (CHB) develop liver cirrhosis every year, and a subset of these individuals suffer from liver failure or HCC. Around 600,000 new HCC cases are diagnosed annually worldwide, with HCC being relatively common in Asia-Pacific countries and sub-Saharan Africa; more than 70 % of HCC patients are diagnosed in Asia (with 55 % in China) [1]. However, HCC is relatively uncommon in the USA, Europe and Australia [1,2]. The majority of HCC develops in patients with cirrhosis, which is most often attributable to chronic HBV infection followed by chronic HCV in the Asia-Pacific region [3].

A recent genome-wide association study (GWAS) using Japanese CHB cases and controls confirmed that 11 SNPs in a region including *HLA-DPA1* and *-DPB1* were associated with CHB [4]. Moreover, a GWAS using chronic HBV carriers with and without HCC in five independent Chinese populations reported that one SNP (rs17401966) in *KIF1B* was associated with HCC susceptibility [5]. In the present study, we performed replication studies using Japanese, Korean and Hong Kong Chinese cases and controls in order to evaluate the reported association with HBV-derived HCC in other East Asian populations.

## Results

We performed SNP genotyping of rs17401966 located in the *KIF1B* gene for the purpose of replication analysis of the previous GWAS report [5]. Four distinct cohorts were used for these replication analyses (Table 1). We first examined two independent Japanese case–control samples including 179 cases and 769 controls from Biobank Japan (replication 1), and 142 cases and 251 controls from various hospitals (replication 2). We did not detect any associations between rs17401966 and HCC in the Japanese cohorts (replication 1: OR = 1.09; 95 % CI = 0.82-1.43, replication 2: OR = 0.79; 95 % CI = 0.54-1.15). We further examined Korean case–control samples comprising 164 cases and 144 controls (replication 3) and Hongkongese 94 HCC cases and 187 CHB controls (replication 4), but again did not detect any association (replication 3: OR = 0.95; 95 % CI = 0.66-1.36, replication 4: OR = 1.17; 95 % CI = 0.79-1.75). Logistic regression analysis adjusted for age and gender also did not show any association (P_log_ = 0.65, 0.27, 0.11, 0.56 for each replication panel). Moreover, we conducted meta-analysis to combine these studies, also not detect any association (P_meta_ = 0.97).

**Table 1:** Association between rs17401966 and HBV-derived HCC

cohort	sample size	cases			controls				OR		
	(cases/controls)	GG	AG	AA	GG	AG	AA	HWE p	(95 % CI)	*P*^a^	*P*_het_^b^
replication 1	179/769	13	61	105	45	261	463	0.599	1.09	0.578	
(Japan 1)		(7.2)	(34.1)	(58.7)	(5.9)	(33.9)	(60.2)		(0.82-1.43)		
replication 2	142/251	5	46	91	14	91	146	1	0.79	0.212	
(Japan 2)		(3.5)	(32.4)	(64.1)	(5.6)	(36.2)	(58.2)		(0.54-1.15)		
replication 3	164/144	17	59	88	15	55	74	0.616	0.95	0.790	
(Korea)		(10.4)	(36.0)	(53.6)	(10.4)	(38.2)	(51.4)		(0.66-1.36)		
replication 4	94/187	10	39	44	13	80	94	0.767	1.17	0.432	
(Hong Kong)		(10.6)	(41.5)	(46.8)	(6.9)	(42.8)	(50.3)		(0.79-1.75)		
Meta-analysis ^c^									0.996	0.965	0.423
									(0.84-1.18)		

^a^P value of fisher’s exact test for allele model.

^b^Result of Breslow-Day test.

^c^Results of meta-analysis were calculated by the Mantel-Haenzel method.

## Discussion and conclusions

Zhang et al. [5] reported that SNP rs17401966 was significantly associated with HBV-related HCC (joint OR = 0.61). They conducted a GWAS using 348 cases and 359 controls in a population in Guangxi in southern China, and selected 45 SNPs for the replication study based on the results (P < 10^-4^). In the first replication study, they used 276 cases and 266 controls from Beijing in northern China, and 5 SNPs showed the same direction of association as in the GWAS (P < 0.05). They performed a further replication study (of 507 cases and 215 controls) in Jiangsu in eastern China and only one SNP showed the same trend (P = 3.9 × 10^-5^). Guangdong and Shanghai samples from southern and eastern China were used for further replication studies. The association yielded a p-value of 1.7 × 10^-18^ on meta-analysis.

We performed four replication analyses using Japanese, Korean and Hong Kong Chinese samples (Table 1). Although sample size of each cohort is smaller than that of the previous GWAS, we conducted mete-analysis of all our study. The result did not show any association between rs17401966 and HBV-derived HCC (P_meta_ = 0.97).

This may be due to differences in genetic diversity among Japanese, Korean and Chinese populations. A maximum-likelihood tree of 126 populations based on 19,934 SNPs showed that Japanese and Korean populations form a monophyletic clade with a 100 % bootstrap value [6]. However, Chinese populations form a paraphyletic clade with two other populations. This indicates that Japanese and Korean populations are genetically closer to one another than the Chinese population.

We did not find any association with Hong Kong Chinese cohort (P = 0.43). Moreover, a study using 357 HCC cases and 354 HBV-positive non-HCC controls in Hong Kong Chinese did not show any significant difference (P = 0.91) [7]. Previous population studies have revealed that various Han Chinese populations show varying degrees of admixture between a northern Altaic cluster and a southern cluster of Sino-Tibetan/Tai-Kadai populations in southern China and northern Thailand [6]. Although Hong Kong is located closed to the Guangdong (cohort 3 of Zhang et al study), there is great heterogeneity for rs17401966 between Hong Kong cohorts (our study and Chan’s study [7]) and Guangdong cohort (our study versus Zhang’s study: P_het_ = 0.0066; Chan’s study versus Zhang’s study: P_het_ = 0.035). This result suggests the existence of other confounding factors, which can differentiate the previous study in China and this study.

One of the possible reasons could be the high complexity of multivariate interactions between the genomic information and the phenotype that is manifesting. HCC development is a multiple process which links to causative factors such as age, gender, environmental toxins, alcohol and drug abuse, higher HBV DNA levels, and HBV genotype variations [8]. The eight HBV genotypes display distinct geographical and ethnic distributions. Genotypes B and C are prevalent in Asia. Specific variations in HBV have been associated with cirrhosis and HCC. These variations include in particular mutations in pre-core region (Pre-C), in basal core promoter (BCP) and in ORF encoding Pre-S1/Pre-S2/S and Pre-C/C. Because there is an overlap between Pre-C or BCP mutations and genotypes, these mutations appear to be more common in genotype C as compared to other genotypes [9].

Aflatoxins are a group of 20 related metabolites and Aflatoxin B1 is the most potent naturally occurring chemical liver carcinogen known. Aflatoxin exposures multiplicatively increase the risk of HCC in people chronically infected with HBV, which illustrates the deleterious impact that even low toxin levels in the diet can have on human health [10-12]. Liu and Wu estimated population risk for aflatoxin-induced HCC around the world [13]. Most cases occur in sub-Saharan Africa, Southeast Asia and China, where populations suffer from both high HBV prevalence and largely uncontrolled exposure to aflatoxin in food. But we could not obtain the information of these confounding factors from both of the previous GWAS study and this study. A much wider range of investigations is thus needed in order to elucidate the differences in HCC susceptibility among these Asian populations.

## Methods

### Samples

Case and control samples used in this study were collected from Japan, Korea and Hong Kong listed in supplementary Additional file 1: Table S1. A total of 179 cases and 769 control subjects were analyzed in the first replication study. DNA samples from both CHB controls and HBV-related HCC cases used in this study were obtained from the BioBank Japan at the Institute of Medical Science, the University of Tokyo [14]. Among the BioBank Japan samples, we selected HBsAg-seropositive CHB patients with elevated serum aminotransferase levels for more than six months, according to the guidelines for diagnosis and treatment of chronic hepatisis from The Japan Society of Hepatology (http://www.jsh.or.jp/medical/gudelines/index.html). The mean (and standard deviation; SD) age was 62.0 (9.4) years for the cases and 54.7 (13.5) years for the controls. The second Japanese replication sample sets for the cases (n = 142) and controls (n = 251) study were obtained from 16 hospitals. The case samples for the second replication included 142 HCC patients and the controls included 135 CHB patients and 116 asymptomatic carriers (ASC). The mean (SD) age was 61.3 (10.2) years for the cases and 56.2 (10.9) years for the controls. The Korean replication samples were collected from Yonsei University College of Medicine. The third replication set was composed of 165 HCC patients and 144 CHB patients. The mean (SD) age was 52.2 (8.9) and 37.3 (11.3) years for the cases and controls, respectively. The samples in Hong Kong were collected from the University of Hong Kong, Queen Mary Hospital. The fourth replication set was composed of 94 HCC patients and 187 CHB patients. The mean (SD) age was 58.0 (10.5) and 56.9 (8.3) years for the cases and controls, respectively. All participants provided written informed consent. This research project was approved by the Research Ethics Committees at the Institute of Medical Science and the Graduate School of Medicine, the University of Tokyo, Yonsei University College of Medicine, the University of Hong Kong, Nationa Center for Global Health and Medicine, Hokkaido University Graduate School of Medicine, Teine Keijinkai Hospital, Iwate Medical University, Saitama Medical University, Kitasato University School of Medicine, Musashino Red Cross Hospital, Kanazawa University Graduate School of Medicine, Shinshu University School of Medicine, Nagoya City University Graduate School of Medical Sciences, Kyoto Prefectural University of Medicine, National Hospital Organization Osaka National Hospital, Kawasaki Medical College, Tottori University, Ehime University Graduate School of Medicine, and Kurume University School of Medicine.

### SNP Genotyping

For the first replication samples, we genotyped rs17401966 using PCR-based Invader assay (Third Wave Technologies, Madison, WI) [15], and for the second, third and fourth replication samples, we used TaqMan genotyping assay (Applied Biosystems, Carlsbad, CA). In the TaqMan SNP genotyping assay, PCR amplification was performed in a 5-μl reaction mixture containing 1 μl of genomic DNA, 2.5 μl of KAPA PROBE FAST qPCR Master Mix (Kapa Biosystems, Woburn, MA), and 40 x TaqMan SNP Genotyping Assay probe (ABI) for this SNP. QPCR thermal cycling was performed as follows: 95°C for 3 min, followed by 40 cycles of 95°C for 15 s and 60°C for 1 min. The SNP call rate of each replication panel was 100 %, 100 %, 99.7 % and 99.6 %.

### Statistical analysis

We performed Hardy-Weinberg equilibrium test for the case and control samples in each replication study. Fisher’s exact test was applied to two-by-two contingency tables for three different genetic models; allele frequency, dominant and recessive model. Odds ratios and confidence intervals were calculated using the major alleles as references. Meta-analysis was conducted using the Mantel-Haenszel method. Heterogeneity among studies was examined by using the Breslow-Day test. Genotype-phenotype association for the SNP rs17401966 was assessed using logistic regression analysis adjusted for age and gender in plink 1.07 (http://pngu.mgh.harvard.edu/~purcell/plink/index.shtml).

## Abbreviations

HB, Hepatitis b; HBV, Hepatitis b virus; HCC, Hepatocellular carcinoma; CHB, Chronic hepatitis b; HCV, Hepatitis c virus; GWAS, Genome-wide association study; ASC, Asymptomatic carrier.

## Competing interests

The authors declare that they have no competing interests.

## Author contributions

Study design and discussion: H.S., N.N., Y.T., Ko.M., M.M., K.T.; sample collection: Y.T., Ko.M., Y.N., S.H.A., K.H.H., J.Y.P., M.F.Y., S.H., J.H.K., K.A., S.M., M.W., M.Ku., Y.A., N.I., M.H., S.K., E.T., Ke.M., Y.I., E.M., M.Ko., K.H., Y.Mu., Y.H., T.I., K.I., M.S., M.M.; genotyping: H.S., Y.M., M.Y., H.M.; statistical analysis: H.S.; manuscript writing: H.S., N.N., Y.T., M.M., K.T. All authors read and approved the final manuscript.

## Pre-publication history

The pre-publication history for this paper can be accessed here:

http://www.biomedcentral.com/1471-2350/13/47/prepub

## Supplementary Material

Additional file 1 **Table S1.** Samples used in this study. Click here for file

## References

[B1] Parkin DM Bray F Ferlay J Pisani P Global cancer statistics, 2002 CA: a cancer journal for clinicians 2005 55 2 74 108 10.3322/canjclin.55.2.74 15761078

[B2] Parkin DM Global cancer statistics in the year 2000 The lancet oncology 2001 2 9 533 543 10.1016/S1470-2045(01)00486-7 11905707

[B3] Marrero CR Marrero JA Viral hepatitis and hepatocellular carcinoma Archives of medical research 2007 38 6 612 620 10.1016/j.arcmed.2006.09.004 17613352

[B4] Kamatani Y Wattanapokayakit S Ochi H Kawaguchi T Takahashi A Hosono N Kubo M Tsunoda T Kamatani N Kumada H A genome-wide association study identifies variants in the HLA-DP locus associated with chronic hepatitis B in Asians Nature genetics 2009 41 5 591 595 10.1038/ng.348 19349983

[B5] Zhang H Zhai Y Hu Z Wu C Qian J Jia W Ma F Huang W Yu L Yue W Genome-wide association study identifies 1p36.22 as a new susceptibility locus for hepatocellular carcinoma in chronic hepatitis B virus carriers Nature genetics 2010 42 9 755 758 10.1038/ng.638 20676096

[B6] Abdulla MA Ahmed I Assawamakin A Bhak J Brahmachari SK Calacal GC Chaurasia A Chen CH Chen J Chen YT Mapping human genetic diversity in Asia Science (New York, NY 2009 326 5959 1541 1545 10.1126/science.1177074 20007900

[B7] Chan KY Wong CM Kwan JS Lee JM Cheung KW Yuen MF Lai CL Poon RT Sham PC Ng IO Genome-wide association study of hepatocellular carcinoma in Southern Chinese patients with chronic hepatitis B virus infection PLoS One 2011 6 12 e28798 10.1371/journal.pone.0028798 22174901 PMC3234276

[B8] Sherman M Hepatocellular carcinoma: epidemiology, surveillance, and diagnosis Semin Liver Dis 2010 30 1 3 16 10.1055/s-0030-1247128 20175029

[B9] Yang HI Yeh SH Chen PJ Iloeje UH Jen CL Su J Wang LY Lu SN You SL Chen DS Associations between hepatitis B virus genotype and mutants and the risk of hepatocellular carcinoma J Natl Cancer Inst 2008 100 16 1134 1143 10.1093/jnci/djn243 18695135 PMC2518166

[B10] Qian GS Ross RK Yu MC Yuan JM Gao YT Henderson BE Wogan GN Groopman JD A follow-up study of urinary markers of aflatoxin exposure and liver cancer risk in Shanghai, People's Republic of China Cancer Epidemiol Biomarkers Prev 1994 3 1 3 10 8118382

[B11] Ross RK Yuan JM Yu MC Wogan GN Qian GS Tu JT Groopman JD Gao YT Henderson BE Urinary aflatoxin biomarkers and risk of hepatocellular carcinoma Lancet 1992 339 8799 943 946 10.1016/0140-6736(92)91528-G 1348796

[B12] Wang LY Hatch M Chen CJ Levin B You SL Lu SN Wu MH Wu WP Wang LW Wang Q Aflatoxin exposure and risk of hepatocellular carcinoma in Taiwan International journal of cancer 1996 67 5 620 625 10.1002/(SICI)1097-0215(19960904)67:5<620::AID-IJC5>3.0.CO;2-W 8782648

[B13] Liu Y Wu F Global burden of aflatoxin-induced hepatocellular carcinoma: a risk assessment Environmental health perspectives 2010 118 6 818 824 10.1289/ehp.0901388 20172840 PMC2898859

[B14] Nakamura Y The BioBank Japan Project Clin Adv Hematol Oncol 2007 5 9 696 697 17982410

[B15] Ohnishi Y Tanaka T Ozaki K Yamada R Suzuki H Nakamura Y A high-throughput SNP typing system for genome-wide association studies Journal of human genetics 2001 46 8 471 477 10.1007/s100380170047 11501945

