# Supplementary material for: No association for Chinese HBV-related hepatocellular carcinoma susceptibility SNP in other East Asian populations
Source: BMC Med Genet. 2012 Jun 19;13:47. doi: 10.1186/1471-2350-13-47 (PMC3407509; doi:10.1186/1471-2350-13-47)
Supplement: Additional file 1 — Table S1. Samples used in this study. [file 1471-2350-13-47-S1.docx]

Table S1 Samples used in this study

| cohort | source | case/control | number of  samples | female (%) | age  (mean +/- SD) |
| --- | --- | --- | --- | --- | --- |
| replication 1 | BioBank Japan | HCC | 179 | 34 (19.0) | 62.0 +/- 9.4 |
| (Japan 1) | BioBank Japan | CHB | 769 | 278 (36.2) | 54.7 +/- 13.5 |
| replication 2 | 16 hospitals | HCC | 142 | 26 (18.3) | 61.3 +/- 10.2 |
| (Japan 2) | 16 hospitals | CHB+ASC | 251 | 100 (39.8) | 56.2 +/- 10.9 |
| replication 3 | Yonsei University | HCC | 165 | 37 (22.4) | 52.2 +/- 8.9 |
| (Korea) | Yonsei University | CHB | 144 | 32 (22.2) | 37.3 +/- 11.3 |
| replication 4 | Queen Mary Hospital | HCC | 94 | 14 (14.9) | 58.0 +/- 10.5 |
| (Hong Kong) | Queen Mary Hospital | CHB | 187 | 28 (15.0) | 56.9 +/- 8.3 |
